# Editorial: Pushing the frontiers of stroke care and research - where are we heading?

**DOI:** 10.3389/fstro.2023.1353566

**Published:** 2024-01-15

**Authors:** Ana Catarina Fonseca

**Affiliations:** Clinica Universitária de Neurologia, Centro de Estudos Egas Moniz, Faculdade de Medicina, Universidade de Lisboa, Lisboa, Portugal

**Keywords:** stroke, advances, treatment, inflammation, research, transient ischaemic attack (TIA), machine learning, rehabilitation

The field of stroke and cerebrovascular disease has grown tremendously in the past few years, thanks to game changing technologies in neuroendovascular thrombectomy, neuroimaging, pre-hospital mobile stroke units, stroke recovery, and more. Specialized stroke centers are proliferating around the globe on different continents and in different countries, telemedicine services are connecting more of the underserved rural and community hospitals, stroke care in lower middle-income countries is emerging, and the number of training programs for clinicians to specialize in stroke continue to increase.

Following the unprecedent development in stroke care and research that have occurred over the past few decades, the field of stroke and cerebrovascular disease is now entering a promising era of new diagnostics, therapeutics, predictive algorithms, and healthcare delivery models. In the Frontiers of Stroke Research Topic “*Pushing the frontiers of stroke care and research - where are we heading?*” leading stroke specialists have taken on the task of analyzing new opportunities and emerging challenges in selected areas of stroke ranging from preclinical research to rehabilitation.

In preclinical research, animal models have had a key role in clarifying complex pathophysiology and providing valuable preclinical data to guide further clinical trials. In their article, Nowak et al. reflect on the different existing animal stroke models that exist and their applications as well as the current and future ethical and organizational challenges associated with their use.

Molecular imaging using positron emission tomography (PET) has been fundamental for understanding the concept of penumbra which has allowed the development of new acute treatments for ischemic stroke. Orhii et al. consider how, in the future, PET will enable further study *in vivo* of biochemical processes underlying ischemic damage at each stage of stroke. An example are PET studies with novel inflammation radiotracers that may identify new biological targets and therefore expand disease monitoring and pharmacologic interventions.

Stroke units, reperfusion treatments and dedicated stroke pathways revolutionized stroke treatment. With mechanical thrombectomy being the standard of care for the treatment of strokes associated with large vessels occlusions, the treatment of medium vessels occlusions (MeVOs) is the new frontier for neuroendovascular interventions. Rodriguez-Calieness et al. review the possible challenges associated with demonstrating the efficacy and safety of MeVos treatment by mechanical thrombectomy in clinical trials. In their comprehensive review, they address the different definitions of MeVo reported in literature, how primary and secondary MeVOs may differ in their presentation and treatment, which devices could be used, and how outcomes should be assessed in this group of patients.

There are high hopes that inflammation could be a new treatment target for ischemic stroke prevention. Gorey et al. provide a helpful review on the pathogenic role of inflammation in stroke and atherosclerosis. The authors examine data from observational and genetic studies as well as completed and ongoing randomized controlled trials of anti-inflammatory agents in stroke and cardiac disease. Blood biomarkers of inflammation that may contribute to risk stratification and patient selection for intensive prevention therapies are also discussed.

Artificial intelligence and machine learning offer unprecedented opportunities to transform healthcare for patients with cerebrovascular disease. Oliveira et al. provides us with a systematic review that analyses the potential and limitations of brain computerized tomography (CT) images as predictors of the outcome of ischemic stroke events. They conclude that although brain CT data contains useful information, for now, the use of this information by models does not always translate into statistically significant improvements in the quality of the predictions.

Rehabilitation is one of the areas of stroke that is expected to see more incremental development in the future. Van de Winckel et al. in their article introduce a new observational scale to assess dependency on others in upper limb performance during daily life activities in adults with stroke. The 5-item upper limb lucerne ICF-based multidisciplinary observation scale (UL-LIMOS) was evaluated with Rasch analysis in 407 adults with acute or subacute stroke the authors demonstrated that UL-LIMOS fit the model without problematic floor or ceiling effects with a sensitivity and specificity above 80%. The scale will need be to be subsequentially externally validated so to be used in clinical practice.

Recently, some discussion has been raised regarding the term transient ischemic attack (TIA). Some argue that the term has become obsolete in our age of modern advanced technology. In an article written by Caplan, he looks back and analyzes the term and its history, its support and detraction, and its potential continued usefulness. Still, to move forward it is important to know the history of where we come from.

## Author contributions

AF: Writing – original draft, Writing – review & editing.

